# The Role of Post-Ingestive Feedback in the Development of an Enhanced Appetite for the Orosensory Properties of Glucose over Fructose in Rats

**DOI:** 10.3390/nu12030807

**Published:** 2020-03-18

**Authors:** Kevin P. Myers, Megan Y. Summers, Elizabeth Geyer-Roberts, Lindsey A. Schier

**Affiliations:** 1 Department of Psychology, Bucknell University, Lewisburg, PA 17837, USA; kmyers@bucknell.edu; 2 Neuroscience Program, Bucknell University, Lewisburg, PA 17837, USA; mys005@bucknell.edu; 3 Department of Biomedical Engineering, Bucknell University, Lewisburg, PA 17837, USA; emgr001@bucknell.edu; 4 Department of Biological Sciences, University of Southern California, Los Angeles, CA 90089, USA

**Keywords:** taste, visceroceptive feedback, flavor–nutrient learning, gut nutrient sensing

## Abstract

The simple sugars glucose and fructose share a common “sweet” taste quality mediated by the T1R2+T1R3 taste receptor. However, when given the opportunity to consume each sugar, rats learn to affectively discriminate between glucose and fructose on the basis of cephalic chemosensory cues. It has been proposed that glucose has a unique sensory property that becomes more hedonically positive through learning about the relatively more rewarding post-ingestive effects that are associated with glucose as compared to fructose. We tested this theory using intragastric (IG) infusions to manipulate the post-ingestive consequences of glucose and fructose consumption. Food-deprived rats with IG catheters repeatedly consumed multiple concentrations of glucose and fructose in separate sessions. For rats in the “Matched” group, each sugar was accompanied by IG infusion of the same sugar. For the “Mismatched” group, glucose consumption was accompanied by IG fructose, and vice versa. This condition gave rats orosensory experience with each sugar but precluded the differential post-ingestive consequences. Following training, avidity for each sugar was assessed in brief access and licking microstructure tests. The Matched group displayed more positive evaluation of glucose relative to fructose than the Mismatched group. A second experiment used a different concentration range and compared responses of the Matched and Mismatched groups to a control group kept naïve to the orosensory properties of sugar. Consistent with results from the first experiment, the Matched group, but not the Mismatched or Control group, displayed elevated licking responses to glucose. These experiments yield additional evidence that glucose and fructose have discriminable sensory properties and directly demonstrate that their different post-ingestive effects are responsible for the experience-dependent changes in the motivation for glucose versus fructose.

## 1. Introduction

Simple sugars, artificial sweeteners, and some D-amino acids are thought to reinforce ingestion through signals generated mainly from a single common receptor in the peripheral gustatory system—the T1R2+T1R3 taste receptor [1,2,3,4,5,6,7,8]. However, a recent series of studies by Schier et al. showed that ingestive exposure to two metabolically distinct sugars, glucose and fructose, produced a strong avidity for the orosensory properties of glucose over those of fructose in rats and mice through a T1R2+T1R3-independent receptor mechanism [9,10]. The sensory, physiological, and/or psychological processes that contribute to this exposure-dependent phenomenon are not well understood.

As the gatekeeper of the body, the mammalian gustatory system is organized to respond to a variety of biologically significant chemicals [11]. While expedient, these hardwired responses are only informative about broad categories of food quality and do not always align with a food’s actual consequences. Thus, to better cope with their particular food environment, rodents, like other animals, benefit from a variety of adaptive strategies. Taste/flavor–nutrient learning is one such mechanism. In this phenomenon, which has been well characterized by Sclafani and colleagues, the orosensory properties of a food are associatively linked to the post-ingestive consequences of that food (e.g., nutrition); this, in turn, modifies the appetitive and consummatory responses that are elicited by the orosensory stimulus [12,13]. For example, rats and mice will come to prefer and consume more of an arbitrary flavor that has been associated with intragastric infusions of glucose (i.e., calories) over a different flavor paired with infusions of water (i.e., no calories) [14,15,16]. Moreover, Sclafani and others have shown that certain sugars, like glucose, condition stronger flavor preferences than other sugars, like fructose, matched for calories [12,17]. The post-ingestive sensory and metabolic effects of glucose appear to be particularly potent stimulators of central reward circuits [18,19,20,21]. Therefore, the ability of rodents to discriminate between glucose and fructose could reflect learning to associate even subtle orosensory differences among the two sugars with their divergent post-ingestive outcomes.

Alternatively, mere exposure to the orosensory properties of these two sugars could enhance the ability to discriminate among them, even without distinct post-ingestive input, through a perceptual-reward learning process. This would require that in addition to their mutual activation of the T1R2+T1R3 taste receptor, either glucose activates an alternative receptor that produces further positive affect or that fructose activates an alternative receptor that leads to diminished affect from a cephalic site of action. According to this model, through repeated exposure to each sugar, rats and mice would come to respond more positively to the orosensory properties of glucose. A third possibility is that dietary exposure to glucose and fructose alters peripheral sensory inputs to bias ingestion towards glucose. Peripheral taste receptor availability and/or activity are regulated by metabolic conditions and diet [22,23]. Theoretically then, this could provide a means through which intake is titrated to need and/or environmental availability at the earliest steps of sensory processing.

Here, we aimed to distinguish between these possibilities and further elucidate the mechanisms involved in shaping this phenomenon, specifically testing whether the differential post-ingestive consequences associatively linked to each sugar are responsible for experience-dependent glucose vs. fructose discrimination. To do this, we took advantage of the electronic esophagus preparation to systematically vary the oral stimulus and post-ingestive stimulus during the exposure phase. One group of rats was intragastrically (IG) infused with fructose while consuming fructose and IG infused with glucose while consuming glucose (“Matched” group; Experiments 1 and 2) in separate single-bottle intake sessions. This group was designed to mimic how rats normally experience the oral and post-ingestive effects of consuming these two sugars. A second group received the opposite contingencies (i.e., oral glucose→IG fructose; oral fructose→IG glucose) (“Mismatched” group; Experiments 1 and 2). As such, this group was given equivalent oral and post-oral exposure to the two sugars but in such a way that oral stimulation was uncoupled from differential post-oral stimulation. A third group (“Control”), included in Experiment 2 only, remained naïve to the orosensory properties of glucose and fructose during the exposure stage, and consumed equicaloric fat emulsions accompanied by IG glucose or IG fructose in alternate sessions. This Control group thus has comparable experience as the Matched group with the post-ingestive effects of glucose and fructose infusions, but without the opportunity to associate these with the oral properties of the sugars. To preclude rats from simply attending to intensity differences among the sugars, all groups were given three isomolar/isocaloric concentrations of each sugar with overlapping intensity ranges in a randomized fashion across successive oral—IG conditioning trials. Affective orosensory evaluation for glucose versus fructose was assessed after conditioning in brief access tests. Finally, microstructural patterns of licking during ad libitum oral consumption of each sugar (no IG infusions) were analyzed from post-conditioning single-bottle intake tests. The post-ingestive consequence learning hypothesis would be supported by finding enhanced attraction to glucose over fructose after training only in Matched rats who experienced glucose consumption reliably coupled with its post-ingestive consequences, and not in Mismatched rats for whom oral experience with glucose and fructose was without differential post-ingestive consequences or Control rats who experienced different IG infusions without glucose and fructose taste experience.

## 2. Experiment 1

### 2.1. Subjects

Experimentally-naïve male Sprague–Dawley rats (*n* = 19) born in the laboratory to breeding stock obtained from Charles River were approximately 65 days old and weighed 344 ± 37 g at the start of the experiment. Rats were housed individually in 8 × 16 × 10.5″ plastic tub cages with corncob bedding in a colony room maintained at approximately 20 °C, with a 12:12 h light:dark cycle (lights on at 0600) throughout the experiment. All procedures were approved by the Bucknell University IACUC and were conducted in accordance with the National Institutes of Health Guide for the Care and Use of Laboratory Animals.

### 2.2. Surgery

Prior to training, each rat was surgically outfitted with a chronic indwelling intragastric (IG) Silastic catheter (1.02 mm ID, 2.16 mm OD) under ketamine/xylazine anesthesia (65 and 10 mg/kg, IP). The catheter was routed from the stomach to the peritoneal cavity and then subcutaneously to exit between the shoulders. The free end of the catheter was attached to a Luer-Lok connector that remained capped when not in use.

### 2.3. Stimuli

Reagent-grade glucose and fructose were prepared fresh with H_2_O as needed. Corn oil (Mazola, ACH Foods Inc.) emulsions were made by mixing (weight: volume) 1.4%, 1.9%, 2.5%, and 4.5% corn oil with 0.6% of an emulsifying agent (sodium stearoyl lactylate) and H_2_O in a standard kitchen blender.

### 2.4. Training Apparatus

Experimental sessions were conducted in 10 identical cylindrical test chambers, 35 cm high × 25 cm diameter, made of opaque plastic with a wire grid floor. When placed in a test chamber, the rat’s IG catheter was attached to infusion tubing suspended overhead on a standard fluid swivel/counterbalance arm assembly, which was, in turn, connected to an individually computer-controlled syringe pump. The drinking bottle was held on a motorized bottle retractor (modified Med Associates ENV-252) so that the sipper tube was accessible through an aperture at the front of the chamber. Licking responses were recorded throughout the session by electronic contact lickometers interfaced to a computer. Additionally, licking activated the interfacing pump to deliver IG infusion at a rate ~1.33 mL/min, adjusted individually for each rat to achieve a 1:1 ratio of IG infusion to volume consumed by mouth.

### 2.5. Oral—IG Training Procedures

After a post-operative recovery of at least eight days, rats were adapted to a restricted feeding schedule (14 g ration fed daily at lights out). Then, rats were acclimated to lick at the sipper spout and receive yoked IG infusions in a daily 30 min intake session with 4.5% corn oil emulsion (oral sipper spout) and water (IG). The first two such sessions were preceded by overnight water restriction to promote consumption. Following this 8 day acclimation period, rats were subdivided into two groups and trained in a series of 18 daily 30 min sessions to associate consumption of either glucose or fructose, with IG infusion of the same or the opposite sugar as follows: in each of the 3 six-day training blocks, rats were given three different concentrations (0.316, 0.56, and 1.1 M) of glucose and of fructose to consume. Rats in the Matched (*n* = 9) always received IG infusions of the same sugar and concentration that they consumed orally, whereas rats in the Mismatched group (*n* = 10) received intragastric infusions of the same concentration of the opposite sugar that they consumed orally. These two groups were determined by random assignment. The order in which each concentration/sugar was presented was randomized in each six-day block. Each training session lasted 30 min, conducted in the middle of the lights-on period. The first nine training sessions were performed after ~22 h of home cage water restriction to stimulate drinking. Daily chow rations (~14 g) were fed one hour after each session.

### 2.6. Brief Access Testing Apparatus

The brief access gustometer (Davis MS 80, Dilog Instruments, Tallahassee, FL, USA) consisted of a Plexiglas cage with a wire grid floor and a stainless steel front wall. Positioned in the center of the stainless steel front wall was an aperture with a computer-controlled shutter that could be opened to allow the rat to access one of several sipper tubes mounted on a motorized tray. The motorized tray was positioned in front of the shutter/access slot prior to the start of each trial, as designated by the computer program. In a typical session, at the start of each trial, the shutter would open, allowing access to a sipper tube. The shutter remained open for 15 s after the rat initiated licking. The shutter would then close for a 7 s inter-trial interval, during which time the motorized tray would position a different sipper tube in front of the access slot. The order in which the sipper tubes were presented was randomized. Animals were free to initiate trials until the 30 min session terminated.

### 2.7. Brief Access Test Procedures

Following the completion of the oral—IG training phase, rats were trained to lick in the Davis Rig as follows: after overnight water restriction, rats were given a single session in which two bottles of water switched position once per minute for 15 min. On the next two training sessions, rats were provided single access to one of 6 sipper bottles containing 4.5% corn oil emulsion. The bottle available at the access slot was switched every 15 s for 30 min. Daily rations and water were given in the home cage following each of these brief access training sessions. In the final test sessions, conducted under food restriction only, 0.316, 0.56, and 1.1 M glucose and fructose (6 solutions) were presented in serial 15 s trials in a randomized fashion (6 solutions/block; randomized within each block without replacement). This test was repeated twice on consecutive days. No IG infusions were made on these brief access training and test sessions.

### 2.8. Licking Microstructure Test Procedures

Following the brief access tests, rats were given single access to 0.56 M glucose and fructose in separate 30 min test sessions in the same apparatus used for oral—IG training. To re-familiarize the rats with single access sessions, rats were given a single 30 min session in which 4.5% corn oil was provided in the sipper bottle. On the next six days, rats were offered 0.56 M glucose or fructose, alternating across days. No IG infusions were made during these test sessions.

### 2.9. Data Analyses

Licks to each sugar concentration were averaged from all trials completed across the two post-conditioning brief access taste tests. These lick scores were then analyzed in 2 (group; Matched vs. Mismatched) × 2 (sugar; glucose vs. fructose) × 3 (concentration, 0.316, 0.56, and 1.1 M) repeated measures ANOVA. The time-stamped lick records from the licking microstructure tests were used to calculate initial burst size, mean burst size/session, and mean session number of licking bursts/session. Bursts were defined as a period of active licking before a ≥ 1.0 s pause active licking [24]. Burst size was determined by summing total licks within a burst (i.e., between two pauses). Initial burst size was calculated as the average number of licks in the first two bursts of the session. These output parameters were averaged across the 3 test sessions for each sugar. Separate 2 (group; Matched vs. Mismatched) × 2 (sugar, glucose vs. fructose) ANOVAs were run on total licks, initial burst size, mean burst size/session, and mean burst number/session. Two rats from the Mismatched group were excluded from these analyses due to catheter failure or signs of illness. For all statistical analyses, a *p* value of ≤0.05 was considered significant. Post-hoc main effects ANOVAs or *t*-tests were used, where appropriate, to break down overall significant main effects and interactions.

## 3. Results

### 3.1. Brief Access Taste Tests

**Figure 1:**
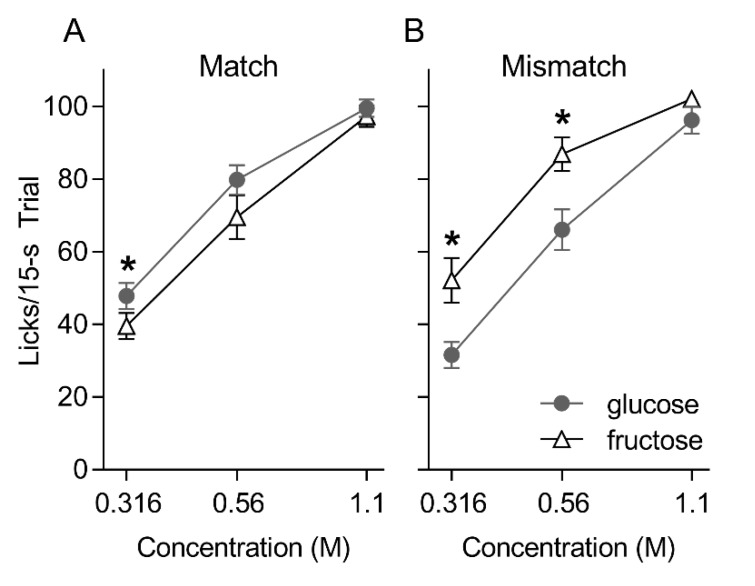
Mean ± SEM licks to three concentrations of glucose and fructose presented in randomized order in 15 s trials for the Matched (**A**) and Mismatched (**B**) training groups during the post-conditioning brief access taste test. Asterisks (*) indicate significant pair-wise differences (glucose versus fructose) at that concentration.

In the post-oral—IG training brief access taste tests, both groups generally licked in a concentration-dependent fashion for glucose and fructose [group: F(1, 17) = 0.004, *p* = 0.95; sugar: F(1, 17) = 2.60, *p* = 0.13; concentration: F(2, 34) = 231.41, *p* < 0.001] (Figure 1). However, whereas the Mismatched group licked significantly more for fructose than glucose at the 0.316 and 0.56 M concentrations (*ps* = 0.003 and 0.013, respectively), the Matched group displayed the opposite pattern and licked more for glucose compared to fructose at the 0.316 M concentration (*p* = 0.04) [group × sugar: F(1, 17) = 17.212, *p* = 0.001; group × sugar × concentration F(2, 16) = 5.699, *p* = 0.007]. Both groups took a comparable numbers of trials on the brief access test [Mean ± SEM trials over two repetitions of the test, Matched = 68.8 ± 4.2; Mismatched = 62.8 ± 5.2, t(17) = 0.88, *p* = 0.39].

### 3.2. Single-Bottle Intake Tests

**Figure 2:**
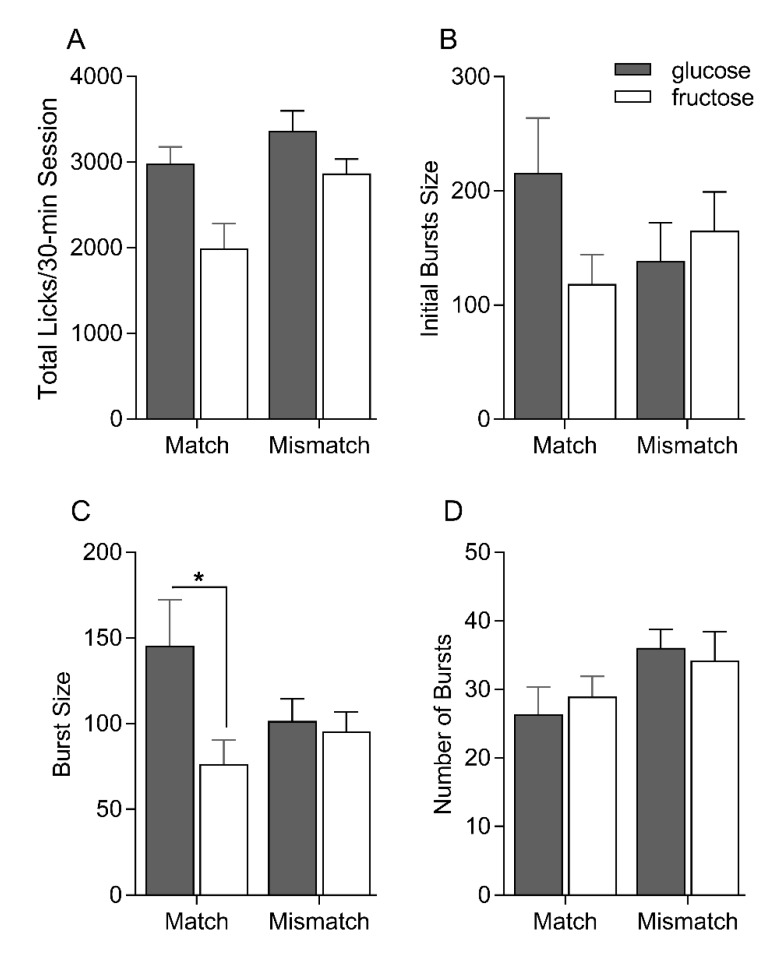
Mean ± SEM total licks (**A**), initial bursts size (**B**), overall burst size (**C**) and overall number of bursts (**D**) on the post-conditioning single-bottle 0.56 M glucose and fructose intake test sessions for Matched and Mismatched groups. Significant differences indicated by asterisks (*).

The total lick counts for each 30 min session demonstrates that both the Matched and Mismatched groups each consumed significantly more glucose than fructose in the single-bottle intake tests [F(1, 15) = 24.3, *p* < 0.001] (Figure 2). Although this difference between glucose and fructose was nominally larger for the Matched than the Mismatched group, the interaction was not statistically significant [F(1, 15) = 2.17, *p* = 0.12]. The Mismatched group consumed more overall than did the Matched group [F(1, 15) = 5.06, *p* < 0.05]. Burst size is thought to reflect the orosensory affective valence of the solution; larger bursts are typically associated with a greater positive valence [24]. Consistent with the brief access test data, the Matched group tended to lick more for glucose relative to fructose in the initial bursts when licking is primarily driven by orosensory input. The Mismatched group, on the other hand, generated a similar number of licks in the initial bursts for glucose and fructose. Nevertheless, no statistical differences between group and/or sugar were found on this initial burst measure [sugar F(1, 15) = 0.16, *p* = 0.70; sugar F(1, 15) = 1.05, *p* = 0.32; group × sugar F(1, 15) = 3.21, *p* = 0.09]. Looking across the entire 30 min intake sessions, both groups displayed larger lick bursts for glucose compared to fructose, but this difference was markedly larger for the Matched group (*p* = 0.02) [sugar F(1, 15) = 6.68, *p* = 0.02; group F(1, 15) = 0.36, *p* = 0.56; group × sugar F(1, 15) = 4.72, *p* = 0.05]. Burst number did not vary by group and/or sugar [sugar F(1, 15) = 0.03, *p* = 0.87; group F(1, 15) = 2.77, *p* = 0.16; group × sugar F(1, 15) = 1.06, *p* = 0.32].

## 4. Experiment 2

The results of Experiment 1 demonstrated that rats given explicit experience consuming glucose and fructose faithfully paired with their respective and distinct post-ingestive effects (Matched group) subsequently displayed a different pattern of licking for the two sugars than did the rats that were given equivalent orosensory experience with glucose and fructose but uncoupled from their distinguishing post-ingestive effects (Mismatched). These results generally accorded with previous studies by Schier et al. [9,10], though we noted a couple exceptions.

First, in previous studies, rats and mice that had extensive experience orally ingesting glucose and fructose in separate sessions later responded very positively to glucose compared to fructose at all three sugar concentrations. Here, the Matched group in Experiment 1, which was designed to correspond to this straightforward sugar discrimination, showed a rather modest avidity for glucose compared to fructose. In this case, because the IG infusions accompanied intake at a 1:1 volume, the amount of sugar reaching the gut was much greater than with regular consumption. Thus, it may be the case that the negative feedback associated with high sugar loads masked differences in the post-ingestive reward value of each sugar. This is supported by prior demonstrations that IG infusions of a more concentrated carbohydrate solution to the gut can be less effective for flavor–nutrient preference conditioning relative to a moderate concentration [25]. Thus, in Experiment 2, we used the same conditioning procedure, but with a lower range of sugar concentrations.

Second, Schier et al. [9] found that naïve or single sugar-exposed rats treated glucose and fructose quite comparably in the post-conditioning brief access taste test. In contrast, here, rats in the Mismatched group licked more for fructose than glucose at the low to mid-range concentrations. This could be based on orosensory experience with sugars that vary in intensity at this concentration range; fructose is thought to be slightly more intense than glucose at isomolar concentrations. Accordingly, Mismatched rats may be simply more attuned to the intensity gradient associated with sugar and respond in a more discriminating manner than naïve rats would. On the other hand, although the Mismatched condition was designed to receive the same post-ingestive stimulus after drinking glucose and fructose (i.e., a blend of the two sugars), it is possible that the orally consumed and IG infused sugars were not mixing at a perfectly matched rate or proportion in the stomach during these training sessions. If that is the case, then one could argue that the Mismatched group received orosensory experience with fructose paired with the relatively more rewarding post-ingestive effects of glucose (and vice versa), which, in turn, established a more positive evaluation of the orosensory properties of fructose. Thus, in this Experiment 2, we also included a control group that remained naïve to the orosensory properties of glucose and fructose during training. Instead, these rats orally consumed calorically-matched concentrations of corn oil emulsion, while receiving the same alternation of glucose and fructose IG infusions as the other groups. As such, this group allows for comparison of the Matched and Mismatched groups’ oral responses to glucose and fructose relative to rats who experience the metabolic effects of the sugars but without experience with their putatively distinct orosensory properties.

### 4.1. Subjects

Twenty-four experimentally-naïve, male Sprague–Dawley rats born in the laboratory to breeding stock obtained from Charles River were maintained under similar conditions to those described in Experiment 1. All rats were approximately 65 days old and weighed 331 ± 22 g at the start of the experiment. Prior to behavioral training and testing, rats had a Silastic IG catheter implanted under ketamine/xylazine anesthesia and were allowed at least 8 days post-operative recovery, as described for Experiment 1.

### 4.2. Training and Testing Procedures

The design and protocol of Experiment 2 were similar to Experiment 1 with the following exceptions. First, the concentrations of glucose and fructose used for training and testing were lowered to 0.179, 0.237, and 0.316 M. Experiment 2 included Matched (*n* = 9) and Mismatched (*n* = 9) groups as described in Experiment 1, as well as an additional control group (Control, *n* = 6). The control group remained naïve to sugar’s orosensory properties throughout training. While the experimental groups consumed glucose and fructose accompanied by IG infusion of the same (Matched) or the opposite (Mismatched) sugar, Control rats consumed corn oil emulsions that were equated on the basis of calories to the sugars consumed by the experimental groups (1.4%, 1.9%, 2.5%). On oral—IG training sessions, Control rats consumed by mouth and received yoked IG infusion of isocaloric glucose and fructose on alternating days. Accordingly, the Control rats received an equivalent number of IG infusions of glucose and fructose as the Matched and Mismatched rats, while remaining completely naïve to the orosensory properties of both sugars. After oral—IG training, all groups were trained and tested for their licking responses to 0.179, 0.237, and 0.316 M glucose and fructose in brief access test sessions (15 s trials; randomized order) as described for Experiment 1. As in Experiment 1, this was followed by single access licking microstructure tests. In this case, rats were given three 30 min sessions with 0.316 M glucose and three 30 min sessions with 0.316 M fructose in alternating order (1 session/day). Data were analyzed as described for Experiment 1, except that a third group (Control) was included in the model.

## 5. Results

### 5.1. Brief Access Taste Tests

**Figure 3:**
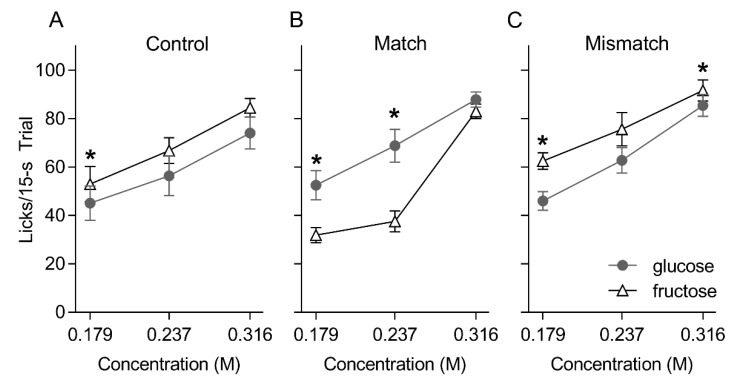
Mean ± SEM licks to three concentrations of glucose and fructose presented in randomized order in 15 s trials for the Control (**A**), Matched (**B**) and Mismatched (**C**) training groups during the post-conditioning brief access taste test. Asterisks (*) indicate significant pair-wise differences (glucose versus fructose).

After oral—IG conditioning, all groups licked in a concentration-dependent fashion for glucose and fructose [group: F(2, 21) = 2.30, *p* = 0.13; sugar: F(1, 21) = 0.10, *p* = 0.76; concentration: F(2, 42) = 151.88, *p* < 0.00001] (Figure 3). However, significant interactions involving group, sugar and concentration revealed group-wise differences in the relative licks to glucose and fructose [group × sugar F(2, 21) = 14.348, *p* < 0.001; group × sugar × concentration F(4, 42) = 4.355, *p* = 0.005]. Namely, the Control and Mismatched groups licked more for fructose compared to glucose at select concentrations (0.179 M: *ps* = 0.02 and 0.01, respectively; 0.237 M: *ps* = 0.09 and 0.16, respectively; 0.316 M: *ps* = 0.07 and 0.04, respectively). By stark contrast, the Matched group licked significantly more for glucose compared to fructose at the 0.179 and 0.237 M concentrations (*ps* = 0.003 and 0.01, respectively). All groups took a comparable number of trials on the brief access tests (Mean ± SEM trials over two repetitions of the test, Matched = 87.3 ± 5.4, Mismatched = 74.9 ± 7.3, Control = 77.2 ± 6.5, F(2, 21) = 1.10, *p* = 0.35).

### 5.2. Single-Bottle Intake Tests

**Figure 4:**
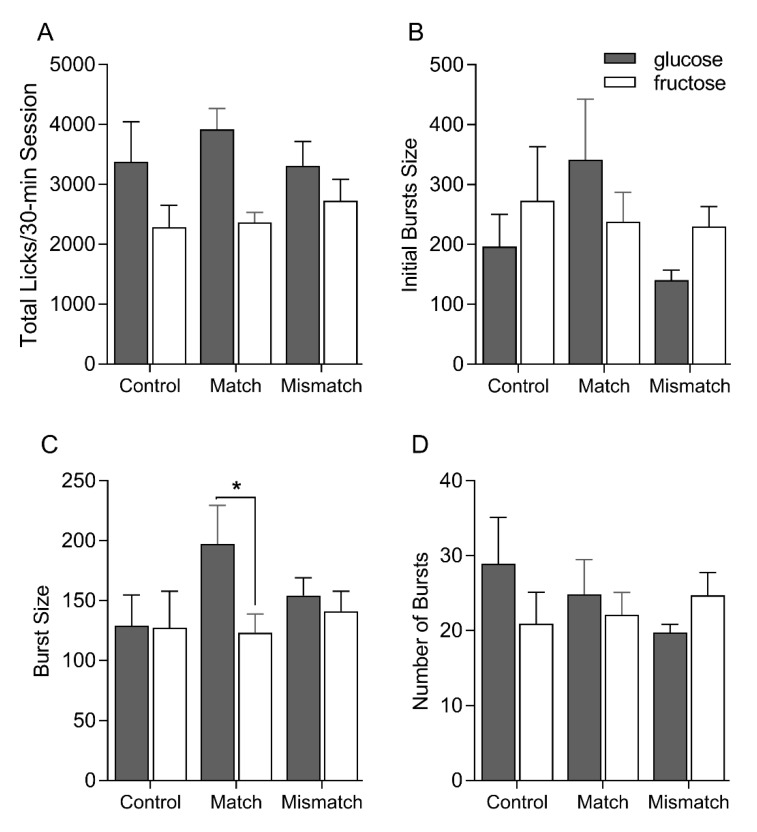
Mean ± SEM total licks (**A**), initial bursts size (**B**), overall burst size (**C**) and overall number of bursts (D) on the post-conditioning single-bottle 0.316 M glucose and fructose intake test sessions for Control, Matched and Mismatched groups. Significant differences are indicated by asterisks (*).

Overall, all groups licked significantly more for glucose than for fructose in these tests [main effect of sugar F(1, 21) = 39.11, *p* < 0.0001] (Figure 4). This effect was most pronounced in the Control and Matched groups, though no significant effects of interactions involving group were found [main effect of group F(2, 21) = 0.17, *p* = 0.84; group × sugar F (2, 21) = 3.08, *p* = 0.07]. Consistent with the brief access licking behavior, initial burst size varied by group, whereby the Matched group tended to lick more for glucose than fructose, while Control and Mismatched groups tended to lick more for fructose. This was supported by a significant group × sugar interaction [F(2, 21) = 4.19, *p* = 0.03; effects of group and sugar [F(2, 21) = 0.94, *p* = 0.41 and F(1, 21) = 0.41, *p* = 0.53, respectively]. The Matched group, but not the Control or Mismatched groups, sustained significantly larger bursts of licking for glucose over fructose across the entire 30 min [main effect of group: F(2, 21) = 0.55, *p* = 0.59; main effect of sugar F(1, 21) = 6.20, *p* = 0.02; group ×sugar F(2, 21) = 3.76, *p* = 0.04]. ANOVA yielded a significant main effect of sugar on burst number [F(1, 21) = 6.47, *p* = 0.02] without an effect or interaction involving group [F(2, 21) = 0.15, *p* = 0.86 and F(2, 21) = 0.51, *p* = 0.67, respectively]. Thus, elevated glucose intake appears to be at least partially accomplished through an increase in bursts of licking for glucose.

## 6. General Discussion

Consistent with previous studies [9,10], we showed that rats can come to respond more positively to the orosensory properties of glucose over those of fructose with ingestive experience. The results further confirm that this is a form of flavor–nutrient learning, whereby even very subtle differences between orosensory stimuli can become behaviorally discriminable through their association with distinct post-ingestive consequences. Licking responses to glucose versus fructose in the brief access trials were dependent upon both the prior oral and IG contingency and total sugar loads. Specifically, rats that received matched oral and IG infusions (glucose→glucose; fructose→fructose) subsequently displayed a heightened avidity for glucose in brief access tests after conditioning with low-mid range sugar concentrations. By contrast, oral exposure to each sugar either paired with the opposite sugar in the gut (Mismatched; glucose→fructose; fructose→glucose) led to small but consistent increases in licking for fructose, compared to glucose in short duration trials, across the concentration ranges tested. Oral→IG conditioning also influenced sugar consumption in 30 min single-bottle intake tests. Rats with matched exposure sustained significantly larger bursts of licking for glucose, compared to fructose. These appetitious effects of glucose were diminished in rats with Control or Mismatched exposure. Collectively, these findings further support a role for flavor–nutrient learning in shaping sugar reward, importantly including the capacity to affectively discriminate among two sugars associated with divergent post-ingestive consequences on the basis of cephalic phase sensory signals.

The nature of the post-ingestive stimuli plays a critical role in establishing the affective discrimination between glucose and fructose. The rather modest avidity for glucose compared to fructose in the brief access test of Experiment 1 was somewhat surprising. Schier et al. [9] had previously shown that rats given the same concentrations (0.316–1.1 M) to consume orally in the conditioning phase, later displayed a robust avidity for glucose compared to fructose in the brief access taste test. We speculated that the ingestion to infusion ratio used here resulted in greater net post-ingestive sugar loads than seen with normal ingestion that, in turn, limited rewarding effects of the post-ingestive stimulation. When sugar content in the gut is too high, the negative post-ingestive effects start to outweigh the positive ones [25]. Therefore, one possibility is that at this relatively high concentration range, the post-ingestive differences between glucose and fructose were minimized, which, in turn, precluded learning about their distinct orosensory properties. Consistent with this, when trained with a lower concentration range (0.179–0.316 M, the Matched group in Experiment 2 subsequently displayed a robust difference in licking for glucose versus fructose in the brief access test, especially at the lower two concentrations. The precise qualitative and quantitative distinguishing features of the post-ingestive stimuli that promote this type of learning now require further investigation.

Both the Control and Mismatched groups displayed small but consistent increases in licks to fructose relative to glucose in the post-conditioning brief access tests. This pattern differs somewhat from the responses shown by naïve rats in previous experiments. Schier et al. [9] found that rats given ingestive exposure to a single sugar, either glucose or fructose (0.316–1.1 M), later licked quite comparably to both sugars in a brief access test, as did the naïve control group. At matched molar concentrations, fructose has been shown to more effectively activate the T1R2+T1R3 taste receptor and first order gustatory neurons, compared to glucose [7,26,27]. We speculated that one possibility was that the Mismatched group from Experiment 1 learned to associate the orosensory properties of fructose with the immediate post-ingestive consequences of the infused glucose before it mixed with fructose in the stomach. However, the fact that the Experiment 2 Control group, who remained naïve to the orosensory properties of sugar during training, displayed a similar licking pattern as the Mismatched group to the two sugars in the final brief access tests suggests that is not likely the case. Alternatively, this could simply reflect subtle differences in responses to the sugars by different groups of rats, as these experiments used rats derived from different sources than those from Schier et al. [9].

Although the Experiment 1 Matched exposure conditions were insufficient to yield robust licking differences for glucose versus fructose in the brief access test, they did not completely disrupt glucose appetition in the single-bottle test at the 0.56 M concentration. Both the Matched and Mismatched groups consumed more glucose than fructose in 30 min tests, but this response was somewhat more pronounced in the Matched group. In particular, the Matched group licked in significantly larger bursts for glucose compared to fructose, indicating the glucose was judged to have greater overall affective value than fructose in this group. The Mismatched group generated similar numbers of licks per burst for the two sugars. Moreover, the Matched group tended to take larger first bursts of glucose when ingestion rate is principally under orosensory control. Considering brief access test trials were capped at 15 s and rats were licking maximally (6–7 licks/second) for the 0.56 M concentrations during this test, it could be that even subtle differences in the affective value of the two sugars was concealed by a ceiling effect in the brief access tests in Experiment 1.

Similar overall patterns were observed in the single-bottle intake test in Experiment 2. Although the differences were somewhat subtle, the fact that glucose continued to be intake stimulating in the two groups that had extensive exposure to the oral and/or post-ingestive effects of the two sugars (Mismatched and Control) was surprising and speaks to the importance of this sugar in the control of intake. That said, prior exposure to glucose and fructose was not without any effect. While the Matched group produced significantly larger bursts for glucose compared to fructose, both the Control and Mismatched groups produced similar size bursts of licking for the two sugars at the 0.316 M concentration, Initial burst sizes were more variable in this experiment, precluding strong conclusions about differences in orosensory-based reward for glucose versus fructose across the three groups during this test.

## 7. Concluding Remarks and Perspectives

The integration of chemosensory and metabolic signals is critical for guiding ingestion towards expedient and beneficial options, particularly in complex food environments. The present findings provide a clear example of how seemingly indiscriminable sensory signals can eventually become dissociable as a result of nutrient learning. As such, these findings underscore the importance of orosensory post-ingestive associative learning in the development of adaptive strategies to identify nutrient sources. In this case, neither oral exposure nor post-oral exposure to the two sugars alone was sufficient to change the relative affective values of glucose and fructose. The central and/or peripheral mechanisms subserving this phenomenon now warrant further investigation.
